# Correction: Surfaceome CRISPR screen identifies OLFML3 as a rhinovirus-inducible IFN antagonist

**DOI:** 10.1186/s13059-021-02534-5

**Published:** 2021-11-15

**Authors:** Hong Mei, Zhao Zha, Wei Wang, Yusang Xie, Yuege Huang, Wenping Li, Dong Wei, Xinxin Zhang, Jieming Qu, Jia Liu

**Affiliations:** 1 Shanghai Institute for Advanced Immunochemical Studies and School of Life Science and Technology, ShanghaiTech University, Shanghai, 201210 People’s Republic of China; 2 Department of Respiratory and Critical Care Medicine, Ruijin Hospital and Institutes of Respiratory Diseases, School of Medicine, Shanghai Jiao Tong University, Shanghai, 200025 China; 3 University of Chinese Academy of Sciences, Beijing, 100049 China; 4 Research Laboratory of Clinical Virology, Ruijin Hospital, Shanghai Jiaotong University School of Medicine, Shanghai, 200025 China; 5 Shanghai Clinical Research and Trial Center, Shanghai, 201210 People’s Republic of China; 6 State Key Laboratory of Respiratory Disease, Guangzhou Medical University, Guangzhou, 510182 Guangdong Province China; 7 Gene Editing Center, School of Life Science and Technology, ShanghaiTech University, Shanghai, 201210 People’s Republic of China; 8 Guangzhou Laboratory, No. 9 XingDaoHuanBei Road, Guangzhou International Bio Island, Guangzhou, 510005 Guangdong Province China

**Correction to: Genome Biol 22, 297 (2021).**

10.1186/s13059-021-02513-w

Following publication of the original article [1], the authors identified an error in the author affiliations presented in additional file 1. The additional file has been updated and published in this correction.

The original article [1] has been corrected.

## Supplementary Information

**Additional file 1: Fig. S1.** Construction of CRISPR genome-wide and surfaceome libraries. **Fig. S2.** Quality analyses of constructed genome-wide and surfaceome CRISPR libraries. **Fig. S3.** Evaluation of surfaceome and genome-wide CRISPR libraries. **Fig. S4.** Validation of the screening results. **Fig. S5.** Determination of gene modification efficiency. **Fig. S6.** Validation of the top 10 hits from surfaceome and genome-wide screens. **Fig. S7.** Construction and validation of single clones of ICAM-1^−/−^, RAB5C^−/−^, OLFML3^−/−^, SLC4A7^−/−^ and ATP6AP1^−/+^ H1-Hela cells. **Fig. S8.** Validation of the effects of ICAM-1, RAB5C and OLFML3 on RV infection, related to Fig. 3. **Fig. S9.** Dissection of the functions of RAB5C and OLFML3 in RV infection. **Fig. S10.** RNA-seq analyses of the effects of RAB5C knockout on RV infection. **Fig. S11.** RNA-Seq analyses of the effects of OLFML3 on RV infection (related to Fig. 4). **Fig. S12.** Bar plots showing RT-qPCR quantification of ISG expression in mock and OLML3−/− cells at 24 h post infection of RV-B14 (a) and RV-A16 (b) at an MOI of 2
