# Supplementary material for: Surfaceome CRISPR screen identifies OLFML3 as a rhinovirus-inducible IFN antagonist
Source: Genome Biol. 2021 Oct 22;22:297. doi: 10.1186/s13059-021-02513-w (PMC8532573; doi:10.1186/s13059-021-02513-w)
Supplement: Supplementary file 2 — Additional file 2: Supplementary Tables. Table S1. Primers for construction of CRISPR library and RNA-Seq analyses. Table S2. Primers for construction of sgRNA plasmid. Table S3. Primers for PCR amplification of sgRNA targeted sites for T7E1 analyses. Table S4. RV-B14-specific or RV universal primers for Taqman RT-qPCR. Table S5. Primers for RT-qPCR. Table S6. siRNA sequences. Table S7. Codon-optimized DNA sequences of OLFML3 gene. Table S8. Codon-optimized DNA sequences of RAB5C gene. [file 13059_2021_2513_MOESM2_ESM.docx]

**Additional file 2: Supplementary tables**

**Surfaceome CRISPR screen identifies OLFML3 as a rhinovirus-inducible IFN antagonist**

Hong Mei^1, †^, Zhao Zha^1, †^, Wei Wang^1, †^, Yusang Xie^2^, Yuege Huang^1,3,9^, Wenping Li^1,3,9^, Dong Wei^6^, Xinxin Zhang^6^, Jieming Qu^2,^ *, Jia Liu^1,3,4,5,7,8^*

^1^Shanghai Institute for Advanced Immunochemical Studies, ShanghaiTech University, Shanghai, 201210, P. R. China

^2^Department of Respiratory and Critical Care Medicine, Ruijin Hospital and Institutes of Respiratory Diseases, School of Medicine, Shanghai Jiao Tong University, Shanghai 200025, China

^3^School of Life Science and Technology, ShanghaiTech University, Shanghai, 201210, P. R. China

^4^Shanghai Clinical Research and Trial Center, Shanghai, 201210, P. R. China

^5^State Key Laboratory of Respiratory Disease, Guangzhou Medical University, Guangzhou, 510182, Guangdong Province, China

^6^Research Laboratory of Clinical Virology, Ruijin Hospital, Shanghai Jiaotong University School of Medicine, Shanghai 200025, China

^7^Gene Editing Center, School of Life Science and Technology, ShanghaiTech University, 201210, P. R. China

^8^Guangzhou Laboratory, No. 9 XingDaoHuanBei Road, Guangzhou International Bio Island, Guangzhou 510005, Guangdong Province, China

^9^University of Chinese Academy of Sciences, Beijing, 100049, China

^†^These authors contributed equally to this work

Correspondence should be addressed to J.L. (liujia@shanghaitech.edu.cn), J.Q. (jmqu0906@163.com)

**Additional file 2: Table S1-S6**

**Table of Content:**

Table S1. Primers for construction of CRISPR library and RNA-Seq analyses.

Table S2. Primers for construction of sgRNA plasmid

Table S3. Primers for PCR amplification of sgRNA targeted sites for T7E1 analyses

Table S4. RV-B14-specific or RV universal primers for Taqman RT-qPCR

Table S5. Primers for RT-qPCR

Table S6. siRNA sequences

Table S7. Codon-optimized DNA sequences of OLFML3 gene

Table S8. Codon-optimized DNA sequences of RAB5C gene

**Table S1. Primers for construction of CRISPR library and RNA-Seq analyses**

| Primers | Sequences |
| --- | --- |
| Lib-F | 5’-gtaacttgaaagtatttcgatttcttggctttatatatcttgtggaaaggacgaaacacc-3’ |
| Lib-R | 5’-actttttcaagttgataacggactagccttattttaacttgctatttctagctc-3’ |
| NGS-F | 5’-tctttccctacacgacgctcttccgatctccgtaacttgaaagtatttcga-3’ |
| NGS-R | 5’-gtgactggagttcagacgtgtgctcttccgatctctttttcaagttgataacggac-3’ |

**Table S2. Primers for construction of sgRNA plasmid**

| Genes | Forward primers | Reverse primers |
| --- | --- | --- |
| ICAM1-1 | caccgtgtccacctctaggacccgg | aaacccgggtcctagaggtggacac |
| ICAM1-2 | caccgtgagattgtcatcatcactg | aaaccagtgatgatgacaatctcac |
| ATP6AP1-1 | caccggtgtcattgtaactcacagg | aaaccctgtgagttacaatgacacc |
| ATP6AP1-2 | caccggacctgggaagcattgaagt | aaacacttcaatgcttcccaggtcc |
| SLC4A7-1 | caccgcaccaaggattatctcccag | aaacctgggagataatccttggtgc |
| SLC4A7-2 | caccgccagcatgactgttccattg | aaaccaatggaacagtcatgctggc |
| RAB5C-1 | caccgtggcccccatgtactatcgg | aaacccgatagtacatgggggccac |
| RAB5C-2 | caccgctggcccccatgtactatcg | aaaccgatagtacatgggggccagc |
| NOP56-1 | caccgcgagagcttctccagtcgtg | aaaccacgactggagaagctctcgc |
| NOP56-2 | caccgagccccaaggatctgcactg | aaaccagtgcagatccttggggctc |
| IGSF3-1 | caccgggaatggtaccggctgacgg | aaacccgtcagccggtaccattccc |
| IGSF3-2 | caccgatacggtacttacgccgagg | aaaccctcggcgtaagtaccgtatc |
| PCBP2-1 | caccgtttgtcaatgatcatagcaa | aaacttgctatgatcattgacaaac |
| PCBP2-2 | caccgtggacggcttgggccggtac | aaacgtaccggcccaagccgtccac |
| TFRC-1 | caccgttgttagtctggaagtagca | aaactgctacttccagactaacaac |
| TFRC-2 | caccgcggagccccagaagacatgt | aaacacatgtcttctggggctccgc |
| OLFML3-1 | caccgccactgttcggtttgccagg | aaaccctggcaaaccgaacagtggc |
| OLFML3-2 | caccgttgggctgtctatgccaccc | aaacgggtggcatagacagcccaac |
| PGS1-1 | caccggatcctgctggcctcaccag | aaacctggtgaggccagcaggatcc |
| PGS1-2 | caccgtgatggcatccctctacctg | aaaccaggtagagggatgccatcac |
| DIEXF-1 | caccgcatgcaggcaatacacatgg | aaacccatgtgtattgcctgcatgc |
| DIEXF-2 | caccgactacccctggactcacatg | aaaccatgtgagtccaggggtagtc |
| UTP23-1 | caccggctgccccgctacctcatgg | aaacccatgaggtagcggggcagcc |
| UTP23-2 | caccgacagctgcgtctcccccatg | aaaccatgggggagacgcagctgtc |
| WDR7-1 | caccgtgccatcactcacatcccag | aaacctgggatgtgagtgatggcac |
| WDR7-2 | caccgagccgcgcagactatcacca | aaactggtgatagtctgcgcggctc |
| ATP6AP2-1 | caccgaagtaccatgttgaaaacca | aaactggttttcaacatggtacttc |
| ATP6AP2-2 | caccgaggagagcggatcccagacg | aaaccgtctgggatccgctctcctc |
| ATP6V0C-1 | caccggggcgacgatgagaccgtag | aaacctacggtctcatcgtcgcccc |
| ATP6V0C-2 | caccgcaagagcggtaccggcattg | aaaccaatgccggtaccgctcttgc |
| DHX33-1 | caccgctgctaccggctctacacgg | aaacccgtgtagagccggtagcagc |
| DHX33-2 | caccgtctgcgtcatagatgcacag | aaacctgtgcatctatgacgcagac |
| ATP6V1H-1 | caccggatgtagcaagaacactgcg | aaaccgcagtgttcttgctacatcc |
| ATP6V1H-2 | caccgacgatgttggagaatatgtg | aaaccacatattctccaacatcgtc |
| TMEM199-1 | caccgctcggccagacccaccacag | aaacctgtggtgggtctggccgagc |
| TMEM199-2 | caccgtccaaggtaagtgcagacga | aaactcgtctgcacttaccttggac |
| EXOSC10-1 | caccgcagcaagctatgatgccctg | aaaccagggcatcatagcttgctgc |
| EXOSC10-2 | caccgcctgaaactctactgcaacg | aaaccgttgcagtagagtttcaggc |

**Table S3. Primers for PCR amplification of sgRNA targeted sites for T7E1 analyses**

| Genes | Forward primers | Reverse primers |
| --- | --- | --- |
| ICAM1-1 | gcccgagctcaagtgtctaa | cactaggccacgcatctgat |
| ICAM1-2 | gctgttcccagtctcggagg | ggggccatctggaaaaacac |
| ATP6AP1-1 | aaggctggttgggtgtttct | acctcctacatcctcacgct |
| ATP6AP1-2 | ggtggttgaggtatgggtgg | cacacagacacgggtcagaa |
| SLC4A7-1 | agggataggaagaattactgtggc | gttcccctggagcagaacat |
| SLC4A7-2 | acgtgtttggtcgtggttttc | tccttggttcccatgtgttca |
| RAB5C-1 | tcctggcttgagggttctct | gcgagtgcaatgacgatgtt |
| RAB5C-2 | tgtgcttggagtaggcttcc | gatgcaatgtccccaaacagg |
| NOP56-1 | cctccaggcagagtaggtgt | aagacatggtgggtcacgaaa |
| NOP56-2 | tcgtgacccaccatgtcttc | ttaccccttgtcttcagggc |
| IGSF3-1 | ggcccagatgctgtctttgg | ttcccttgtgagtctgaaacgc |
| IGSF3-2 | ggtgattcctgtgttcccct | caggacccactgcaggtaaa |
| PCBP2-1 | cccctttttgggaagtgtggt | gcgagtctcaaggtccagtg |
| PCBP2-2 | catggacatctcccttgatgaga | agaccaccatccagaatctcc |
| TFRC-1 | ccttgaaccaaaatgttttcccc | cagcagaaacagaaaatgacagc |
| TFRC-2 | aaagtcacagggctgtgtaaa | ccctgtattaaaagctgctgcc |
| OLFML3-1 | gttcctgctggaggcatctaa | gtgtctgctgtcaagccgta |
| OLFML3-2 | cagtattcccagcagagggg | agctcctcaaacctcctcct |
| PGS1-1 | gcccctgacacctggattta | cttgaggagatgaatgctcgc |
| PGS1-2 | cttaatggccgtgttgcatt | tggggcaacaaaaacaagagc |
| DIEXF-1 | cagccagttcctatctggtcc | ggctcattaaggtcccatcca |
| DIEXF-2 | ccaggagggccagttttagc | ggagtgggttacctggcatc |
| UTP23-1 | tgcgtgaggcgtttactgat | acgggtacactttggataccg |
| UTP23-2 | ttcttccgcaacaacttcgg | actgtgaggcttgcagagttt |
| WDR7-1 | gaatgtggactatggtggggg | aggacctagagcattttccca |
| WDR7-2 | ctttcctcccaactcccaagt | agtttccatcctttccgtcgaa |
| ATP6AP2-1 | accatcagtgcaagtgtcgt | tccataacacgagctattctaaatg |
| ATP6AP2-2 | aggactcagcattttgaccagt | cagaagccaagcttgaagcac |
| ATP6V0C-1 | atgtcagtcctctcttctcgc | tccatccaggaagtctcagc |
| ATP6V0C-2 | cccaagacctttggtggctt | gctgatgtcgtcattcaggga |
| DHX33-1 | ctgcggagcagtctggtttt | tccatttgttgttgtttgtctcc |
| DHX33-2 | ttggtgaccacctccgaatg | agatgctttcacgctcctgg |
| ATP6V1H-1 | tctgtgcctactaccatccct | tcctatgggaacggacaggtt |
| ATP6V1H-2 | tgcttagtgctcagtccacc | gcaaccggcctatactactca |
| TMEM199-1 | gacagctaaagtcacctgcg | cgggcaactagttctgggtt |
| TMEM199-2 | agatgggtactggcagccta | ctggggtacagagctggttg |
| EXOSC10-1 | gcttggtgtcgcaaagaaca | acaggcttgctaagttggga |
| EXOSC10-2 | atgggacagcggagactacc | tttcccacctatggaaggtca |

**Table S4. RV-B14-specific or RV universal primers for Taqman RT-qPCR**

| Genes | Sequence |
| --- | --- |
| RV-B14-F | ctagcctgcgtgg |
| RV-B14-R | aaacacggacacccaaagt |
| RV-B14-probe | FAM-tcctccggcccctga-MGB-NFQ |
| RV-general-F | gtgaagagccscrtgtgct |
| RV-general-R | gctscagggttaaggttagcc |
| RV-general-probe | fam-tgagtcctccggcccctgaatg-BHQ1 |

Note: Degenerate codons are R for A or G and S for C or G.

**Table S5. Primers for RT-qPCR**

| Genes | Forward primers | Reverse primers |
| --- | --- | --- |
| IFIT1 | caatactgggtacgcaatcacc | agctcgttttaggacgtgcag |
| IFIT2 | gacacggttaaagtgtggagg | tccagacggtagcttgctatt |
| IFIT3 | aaaagcccaacaacccagaat | cgtattggttatcaggactcagc |
| OAS1 | tgtccaaggtggtaaagggtg | ccggcgatttaactgatcctg |
| OAS2 | ctcagaagctgggttggtttat | accatctcgtcgatcagtgtc |
| OAS3 | gatgtgctgccagcctttga | tgtagctctgtgaagcaggtgga |
| ISG15 | cgcagatcacccagaagatt | gcccttgttattcctcacca |
| ISG20 | ctcgttgcagcctcgtgaa | cgggttctgtaatcggtgatctc |
| IFNB  STAT1  STAT2  SOCS3  OLFML3 | gagctacaacttgcttggattc  cagcttgactcaaaattcctgga  ccagctttactcgcacagc  cctgcgcctcaagaccttc  cgccgactagctgctttagag | caagcctcccattcaattgc  tgaagattacgcttgcttttcct  agccttggaatcatcactccc  gtcactgcgctccagtagaa  cgctccagacgatccactc |

**Table S6. siRNA sequences**

| Genes | Sense sequences (5’-3’) | Anti-sense sequences (5’-3’) |
| --- | --- | --- |
| SOCS3-1  SOCS3-2 | accuuucugauccgcgacatt  gccuauuacaucuacuccgtt | ugucgcggaucagaaaggutt  cggaguagauguaauaggctt |

**Table S7. Codon-optimized DNA sequences of OLFML3 gene**

| **OLFML3-myc-flag** |
| --- |
| atgggccctagcacgccattactgatcctgttcctgctgagctggagcggccctctgcagggccagcagcaccacctggtggagtacatggagagaagactggccgccctggaggagagactggcccagtgccaggaccagagcagcagacacgccgccgagttaagggatttcaagaataaaatgttgcctctgctggaggtggccgagaaggagagagaggccctgagaaccgaggccgacaccatcagcggcagagtggacagactggagagagaggtggactacctggagacccagaatcctgccctgccttgcgtggagttcgacgagaaggtgaccggcggccctggcaccaagggcaagggcagaagaaatgagaagtacgacatggtgaccgactgcggctacaccatcagccaggtgagaagcatgaagatcctgaagagattcggcggccctgccggcctgtggaccaaggaccctctgggccagaccgagaagatctacgtgctggacggcacccagaatgacaccgccttcgtgttccctagattgcgagatttcactttagccatggccgccagaaaggccagcagagtgagagtgcctttcccttgggtgggcaccggccagctggtgtacggcggcttcctgtacttcgccagaagacctcctggcagacctggcggcggcggcgagatggagaataccctgcagctgatcaagttccatctggccaatagaaccgtggtagacagcagcgtgttccctgccgagggcctgatccctccttacggcctgaccgccgacacctacatcgacctggccgccgacgaggagggcctgtgggccgtgtacgccaccagagaggacgacagacacctgtgcctggccaagctggaccctcagaccctggacaccgagcagcagtgggacactccgtgtcctagagagaatgccgaggccgccttcgtgatctgcggcaccctgtacgtggtgtacaataccagacctgccagcagagccagaatccagtgcagcttcgacgccagcggcaccctgactcccgaaagagccgccctgccttacttccctagaagatacggcgcccacgccagcctgagatacaatcctagagagagacagctgtacgcctgggacgacggctaccagatcgtgtacaagctggagatgagaaagaaggaggaggaggtgacgcgtacgcggccggagcagaaactcatctcagaagaggatctggcagcaaatgatatcctggattacaaggatgacgacgataaggtttaa |

**Table S8. Codon-optimized DNA sequences of RAB5C gene**

| **RAB5C-myc-flag** |
| --- |
| atggagctgagctggagaagccctagccctctgagcgccagcctgcacagtactagtccacatccacatgctttatggactacaacggcagggcgtgctatggcaggaagaggaggcgcggcgagacctaatggccctgccgctggtaacaagatatgccagttcaagctggtgctgctgggcgagagcgccgtgggcaagagcagcctggtgctgagattcgtgaagggccagttccacgagtaccaggagagcaccatcggcgccgccttcctgacccagaccgtgtgcctggacgacaccaccgtgaagttcgagatctgggacaccgccggccaggagagataccacagcctagcgccaatgtattacagaggcgcccaggccgccatcgtggtgtacgacatcaccaataccgacaccttcgccagagccaagaattgggtgaaggagctgcagagacaggccagccctaatatcgtgatcgccctggcgggcaataaggctgatctggccagcaagagagccgtggagttccaggaggcccaggcctacgccgacgacaatagcctgctgttcatggagaccagcgccaagaccgccatgaatgtgaatgagatcttcatggccatcgccaagaagctgcctaagaatgagcctcagaatgccaccggcgctccgggacgcaacagaggcgtggacctgcaggagaataatcctgccagcagaagccagtgctgcagcaatacgcgtacgcggccggagcagaaactcatctcagaagaggatctggcagcaaatgatatcctggattacaaggatgacgacgataaggtttaa |
