# Supplementary material for: Surfaceome CRISPR screen identifies OLFML3 as a rhinovirus-inducible IFN antagonist
Source: Genome Biol. 2021 Oct 22;22:297. doi: 10.1186/s13059-021-02513-w (PMC8532573; doi:10.1186/s13059-021-02513-w)
Supplement: Supplementary file 13 — Additional file 13. Review history. [file 13059_2021_2513_MOESM13_ESM.docx]

Review History

**First round of review**

**Reviewer 1**

**Are you able to assess all statistics in the manuscript, including the appropriateness of statistical tests used?** Yes, and I have assessed the statistics in my report.

**Comments to author:**

In this manuscript, the authors performed genome-wide and surfaceome CRISPR screen for RV host factors. They presented the OLFML3 antagonizes type I IFN signaling in an SOCS3-dependent mechanism that promoted the infection. These results have been reliably proven and sound interesting.
My major concern:
1.     The authors sought to validate the top 10 hits, why not used all the enriched sgRNA using false discovery rate (FDR) for the overlap of genome-wide and surfaceome CRISPR screen?
2.     The authors shown the reduced RV-B14 infection rates when knockout of IGSF3, RAB5C and OLFML3, why they focus on RAB5C and OLFML3 in the following study? The role of IGSF3 should be validated in the revised manuscript.
3.     They indicated that RAB5C may be a key regulator of the endosomal release of RV genome, why not use RNA-Seq (The same as used for the OLFML3) to elucidate the mechanism of RAB5C.
My minor concern:
1.     They indicated more enriched sgRNA when using genome-wide screen compared with surfaceome screen (P6), thus I suggest they should compare and discuss the advantages and disadvantages of these two approaches in detail.
2.     P3, line47.  The novel host factors of RVs should be listed in detail.
3.     P7, line 41.  They should use the single clones of gene knockout cells, the same as the ICAM-1-/-, RAB5C-/- and OLFML3-/- cells they used in this study (P9, Line1).
In addition, the gene disruption efficiency is evaluated by T7E1 assay, which is not a reliable method. They should use the Sanger sequencing, Deep Sequencing, and Western blotting if the antibody is available.
4.     The statistical analysis should be added in Fig. S6.
5.     Fig.S7c. Why the band of rescued cells and RAB5C-/- is bigger than the WT?
6.     The RNA-Seq data should be validated by RT-Qpcr.

**Reviewer 2**

**Are you able to assess all statistics in the manuscript, including the appropriateness of statistical tests used?** Yes, and I have assessed the statistics in my report.

**Comments to author:**

In this study authors constructed genome-wide and targeted surfaceome CRISPR screens in parallel to screen for RV virus host factors. The Surfaceome screen purportedly outperformed the genome-wide screen in the success rate of hit identification, and the authors selected a small number of hits to validate. Unfortunately there are serious technical concerns with the analysis and conclusions that render this manuscript unpublishable in its current form.

The first is that the premise of the first part of the paper - that the targeted surfaceome screen outperformed the whole-genome screen - lacks any rigor in its controls. There is no demonstration that either screen performed well using internal technical controls. For example, prior to RV treatment, sequencing the untreated cells would reveal the degree to which the library/screen differentiated essential from nonessential genes. It is not clear whether these screen-level controls were even included in the surfaceome design. Additionally, the authors only applied the Mageck pipeline, which is known to be extremely conservative and not call hits in high noise environments such as a genome-scale screen. Finally, even with Mageck, a strict FDR threshold was used, and no effort was demonostrated to show whether the results are robust to choice of FDR threshold.

The second, and critical, flaw of this paper is that the focus on OLFML3 is predicated on weak CRISPR screen hits and then is "validated" by weak follow up results. The authors describe OLFML3 as encoding a secreted protein. Secreted proteins are invisible to CRISPR screens because knockout of a secreted protein in one cell leaves all the others fully capable of expressing and secreting the protein. The only exception is genes involved in autocrine signaling; e.g. WNT proteins that activate FZD receptors on the same cell. If the protein is secreted, and operates in the pathway indicated, OLFML3-/- cells will show a different phenotype than OLFML3ko in a pooled screen.

Among the other issues herein:

Pg. 3.
Re: "It has been shown that cell proliferation and cell cycle-related proteins such as p53 can perturb the outcome of genome-wide CRISPR screen by masking the positive hits [20]."
Authors should be aware that this statement derived from the cited study (Haapaniemi et al. 2018) has been addressed by other groups (Brown et al. 2019, Bowden et al. 2020, etc.), and is not used as a standard in CRISPR-based functional screening.
From Brown et al. 2019: "While we do not disagree that CRISPR may evoke a p53 response in certain genotypes, examination of hundreds of published genome‐wide CRISPR screens conducted independently by multiple laboratories in different geographic locations showed that major concerns are not warranted when performing CRISPR screens in wild‐type TP53 cells."


Pg. 8.
Re: "These results suggested that except for ICAM-1 the top 10 candidates identified by genome-wide screen were most likely false positive hits."
Two of those 10 genes were previously reported as host dependency factors for influenza A virus - TMEM199, and WDR (Li et al, 2020).

Pg. 15.
Re: "It is surprising to find that compared with genome wide CRISPR screen, surfaceome screen is not only more efficient in identifying cell surface host factors but also exhibits an overall higher positive/success rate."
This statement is unsupported by the data.

Pg. 18
Re: "sgRNAs were ranked by on-target scores and the top 12 sgRNAs were calculated using Rule Set 2 [29]. If less than 12 sgRNAs were obtained, the cutoff of off-target scores was increased sequentially to 40, 60, 80 and 100 until 12 sgRNAs were obtained."
Given that not all guides are from the same bin, are any of the reported 'significant genes' targeted with guides from different bins or are all 10 genes (in surfaceome library) targeted with guides from the same bin (i.e., defined by Rule Set 2)? Authors might consider including some infographics about how many genes have 12 guides with a set cut-off and a varying cut-off and show if and how those impact the hit calling. This would a great addition for the authors' efforts to exclude the possibility that the differential hits in genome wide and surfaceome screens were due to the effects of different number of sgRNAs.

**Authors Response**

**Point-by-point responses to the reviewers’ comments:**

**Reviewer #1: Comments to the Author：**
In this manuscript, the authors performed genome-wide and surfaceome CRISPR screen for RV host factors. They presented the OLFML3 antagonizes type I IFN signaling in an SOCS3-dependent mechanism that promoted the infection. These results have been reliably proven and sound interesting.

**My major concern:**

1. The authors sought to validate the top 10 hits, why not used all the enriched sgRNA using false discovery rate (FDR) for the overlap of genome-wide and surfaceome CRISPR screen?

**Response: We thank the reviewer for raising this interesting point. We initially sought to separately analyze the genome-wide and surfaceome libraries for comparison purpose. Evaluation of the overlapped hits from both libraries, as suggested by the reviewer, will certainly reveal useful information. Because surfaceome library only contains surface proteins, we thus analyzed the overlapped surface proteins from both libraries and the results are included in Additional file: Fig. S4c. Consistent with the previous data, it was found that genome-wide and surfaceome screens can identify different sets of surface proteins, supporting our previous conclusion that surfaceome screen could be used as an alternative screening approach.**

2. The authors shown the reduced RV-B14 infection rates when knockout of IGSF3, RAB5C and OLFML3, why they focus on RAB5C and OLFML3 in the following study? The role of IGSF3 should be validated in the revised manuscript.

**Response: We thank the reviewer for raising this point. We have constructed single clone for IGSF3 knockout for validation experiments. It wasfound that IGSF3 knockout could reduce viral loads in cell lysates and medium supernatants, but did not affect viral entry. These results have been included in Fig 2d-e and Additional file 1: Fig. S9e-f.**

3. They indicated that RAB5C may be a key regulator of the endosomal release of RV genome, why not use RNA-Seq (The same as used for the OLFML3) to elucidate the mechanism of RAB5C.

**Response: As suggested by the reviewer, we have performed RNA-Seq on wild type and RAB5C^-/-^ cells in the absence and presence of RV-B14 respectively. Gene ontology (GO) analyses of differentially expressed genes (DEG) upon RV-B14 infection between WT and RAB5C^-/-^ cells revealed an important role of RAB5C in viral infection and immune response. These results have been included in Additional file 1: Fig. S10.**

**My minor concern:**

1. They indicated more enriched sgRNA when using genome-wide screen compared with surfaceome screen (P6), thus I suggest they should compare and discuss the advantages and disadvantages of these two approaches in detail.

**Response: We appreciate the reviewer’s suggestion and have revised the discussion section to compare the advantages and disadvantages of genome-wide and surfaceome libraries in detail.**

2. P3, line47. The novel host factors of RVs should be listed in detail.

**Response: We have revised the text to include the novel host factors of RV identified from previous studies.**

3. P7, line 41. They should use the single clones of gene knockout cells, the same as the ICAM-1-/-, RAB5C-/- and OLFML3-/- cells they used in this study (P9, Line1).

**Response: As suggested by the reviewer, we have constructed single clones for IGSF3, SLC4A7 and ATP6AP1 (Additional file 1: Fig. S7). In consistency with the bulk population, single clones of IGSF3, SLC4A7 and ATP6AP1 knockout showed resistance to RV infection (Fig. 2d-e).**

In addition, the gene disruption efficiency is evaluated by T7E1 assay, which is not a reliable method. They should use the Sanger sequencing, Deep Sequencing, and Western blotting if the antibody is available.

**Response: We thank the reviewer for raising this point. We have performed Sanger sequencing to determine the knockout efficiency (Additional file 1: Fig. S5 and Additional file 11) in addition to T7E1 assay. Sanger sequencing and T7E1 results are generally consistent and the knockout efficiency was determined to be in the range of 30-90%.**

4. The statistical analysis should be added in Fig. S6.

**Response: We have included the statistical analyses in Additional file 1: Fig. S6b.**

5. Fig.S7c. Why the band of rescued cells and RAB5C-/- is bigger than the WT?

**Response: In the overexpression group, RAB5C contains flag and myc tags at the C-terminus and thus may be different from wild-type RAB5C in molecular weight and charges that may affect its migration rate on SDS-PAGE. The sequence of overexpressed RAB5C gene is included in Additional file 2: Table. S8.**

6. The RNA-Seq data should be validated by RT-Qpcr.

**Response: We apologize for causing the confusion. The heat map results from Fig 4g-h were in fact derived from RT-qPCR. These results were presented as heat map for concision purpose. The corresponding original RT-qPCR results have now been included Additional file 1: Fig S12 and presented as bar plots.**

**Reviewer #2**: Surfaceome CRISPR Screen Identifies OLFML3 as a Rhinovirus-inducible IFN Antagonist
Hong Mei, Zhao Zha, Wei Wang, Yusang Xie, Yuege Huang, Wengoing Li, Dong Wei, Xinxin Zhang, Jia Xie, Jieming Qu, Jia Liu

In this study authors constructed genome-wide and targeted surfaceome CRISPR screens in parallel to screen for RV virus host factors. The Surfaceome screen purportedly outperformed the genome-wide screen in the success rate of hit identification, and the authors selected a small number of hits to validate. Unfortunately there are serious technical concerns with the analysis and conclusions that render this manuscript unpublishable in its current form.

The first is that the premise of the first part of the paper - that the targeted surfaceome screen outperformed the whole-genome screen - lacks any rigor in its controls. There is no demonstration that either screen performed well using internal technical controls. For example, prior to RV treatment, sequencing the untreated cells would reveal the degree to which the library/screen differentiated essential from nonessential genes. It is not clear whether these screen-level controls were even included in the surfaceome design.

**Response: We thank the reviewer for raising this critical point. Like most CRISPR screen studies, we included non-targeting sgRNAs as internal technical controls (Fig 1e). These non-targeting sgRNAs were included in both surfaceome and genome-wide libraries in the presence and absence of RV. As suggested by the reviewer, we have now included the results of the analyses of the shift of essential and non-essential genes. It has been found that non-targeting sgRNA or non-essential genes have distinct fold changes from essential genes (Additional file 1: Fig. S3a).** **Receiver operating characteristic (ROC) analysis have shown that both genome-wide and surfaceome screens can efficiently distinguish essential genes from non-essential genes and non-targeting sgRNA (Additional file 1: Fig. S3b).**

Additionally, the authors only applied the Mageck pipeline, which is known to be extremely conservative and not call hits in high noise environments such as a genome-scale screen. Finally, even with Mageck, a strict FDR threshold was used, and no effort was demonostrated to show whether the results are robust to choice of FDR threshold.

**Response: We agree with the reviewer that MAGeCK has limitations for certain applications and that we did not intend to test an exhaustive list of enriched sgRNAs due to practical reasons. Perhaps the best approach to tackle the concerns with MAGeCK and the choice of FDR is to re-analyze the sgRNA data with a different algorithm. MAGeCK analysis is based on robust rank aggregation (RRA) (Li *et al.*, *Genome Biol*, 2014). We chose to use CB^2^ (Jeong *et al.*, *Genome Res*, 2019), which is based on beta-binomial distribution, to re-analyze the data. A considerable fraction of the top 10 hits from MAGeCK analysis was also identified by CB^2^ (Additional file 1: Fig. S3a-b). These results justified the use of MAGeCK analysis and the choice of FDR.**

The second, and critical, flaw of this paper is that the focus on OLFML3 is predicated on weak CRISPR screen hits and then is "validated" by weak follow up results. The authors describe OLFML3 as encoding a secreted protein. Secreted proteins are invisible to CRISPR screens because knockout of a secreted protein in one cell leaves all the others fully capable of expressing and secreting the protein. The only exception is genes involved in autocrine signaling; e.g. WNT proteins that activate FZD receptors on the same cell. If the protein is secreted, and operates in the pathway indicated, OLFML3-/- cells will show a different phenotype than OLFML3ko in a pooled screen.

**Response: We agree that identification of secretory proteins from CRISPR screen is rare. We thank the reviewer for raising this critical point and have revised the text to better clarify this.**

**However, we believe that our results and conclusion are supported by the experiments we performed. First, the effects of OLFML3 on RV infection is validated by classical virological assays from different aspects, such as viral loads, cytopathic effects, infection rates (IF staining) and others. In addition, the role of OLFML3 is confirmed with clinical isolates of RV. Second, the cellular localization of OLFML3 has not yet been experimentally defined. It is possible that OLFML3 is localized on cellular matrix because the information of cell surface protein included in the surfaceome library is derived from mass spectrometry studies analyzing the cell membrane components. If this is the case, then OLFML3 will not likely to function as paracrine factors affecting neighboring cells. Unfortunately, we did not find any commercial suppliers providing OLFML3 antibodies suitable for immunofluorescence study. Third and most importantly, OLFML3 may be involved in classical autocrine signaling pathway of IFN-IFN α/β receptor (IFNAR), as we illustrated in Fig. 6. As the reviewer pointed out, CRISPR screen is capable of identifying genes involving in autocrine signaling. In fact, this may just be the case with OLFML3. The RNA-Seq results indicated that OLFML3 was involved in the IFN signaling. Autocrine IFN signaling, or so-called positive feedback loop (Fig. 6), exists in the IFN-IFNAR pathway. Taken together, identification of OLFML3 from our surfaceome screen is a surprising, yet understandable, result.**

Among the other issues herein:

Pg. 3.
Re: "It has been shown that cell proliferation and cell cycle-related proteins such as p53 can perturb the outcome of genome-wide CRISPR screen by masking the positive hits [20]."
Authors should be aware that this statement derived from the cited study (Haapaniemi et al. 2018) has been addressed by other groups (Brown et al. 2019, Bowden et al. 2020, etc.), and is not used as a standard in CRISPR-based functional screening.
From Brown et al. 2019: "While we do not disagree that CRISPR may evoke a p53 response in certain genotypes, examination of hundreds of published genome‐wide CRISPR screens conducted independently by multiple laboratories in different geographic locations showed that major concerns are not warranted when performing CRISPR screens in wild‐type TP53 cells."
**Response: We thank the reviewer to bring in this point and have revised the text correspondingly.**

Pg. 8.
Re: "These results suggested that except for ICAM-1 the top 10 candidates identified by genome-wide screen were most likely false positive hits."
Two of those 10 genes were previously reported as host dependency factors for influenza A virus - TMEM199, and WDR (Li et al, 2020).
**Response: We thank the reviewer for raising this point and have revised the text to clarify that our results and conclusion are specific to rhinovirus (picornaviridae family), but not other viruses such as influenza A virus (orthomyxoviridae family).**

Pg. 15.
Re: "It is surprising to find that compared with genome wide CRISPR screen, surfaceome screen is not only more efficient in identifying cell surface host factors but also exhibits an overall higher positive/success rate."
This statement is unsupported by the data.

**Response: We have revised the discussion section for better accuracy.**

Pg. 18
Re: "sgRNAs were ranked by on-target scores and the top 12 sgRNAs were calculated using Rule Set 2 [29]. If less than 12 sgRNAs were obtained, the cutoff of off-target scores was increased sequentially to 40, 60, 80 and 100 until 12 sgRNAs were obtained."
Given that not all guides are from the same bin, are any of the reported 'significant genes' targeted with guides from different bins or are all 10 genes (in surfaceome library) targeted with guides from the same bin (i.e., defined by Rule Set 2)? Authors might consider including some infographics about how many genes have 12 guides with a set cut-off and a varying cut-off and show if and how those impact the hit calling. This would a great addition for the authors' efforts to exclude the possibility that the differential hits in genome wide and surfaceome screens were due to the effects of different number of sgRNAs.

**Response: We appreciate the reviewer for raising this point. Indeed, there is minor difference between the distribution of sgRNA scores between genome-wide and surfaceome libraries. For on-target scores of sgRNAs of the top 10 hits, 100% and 94.2% sgRNAs in the genome-wide and surfaceome libraries respectively have values between 0.6 and 1.0. For off-target scores of sgRNAs of the top 10 hits, 100% and 95.8% sgRNAs in the genome-wide and surfaceome libraries respectively have values below 20. As suggested by the reviewer, an infographics figure has been included in Additional file 1: Fig. S4e to illustrate this**

**Second round of review**

**Reviewer 1**

They have provided the evidence or results and made a revised manuscript accordingly.

**Reviewer 2**

Mei et al considerably revise this work. There is a much improved quality control of the CRISPR screens, suggesting that their screens do provide some interesting information. This statement: “we found that genome-wide and surfaceome screens identified different sets of hit genes for surface proteins (Additional file 1: Fig. S4c), thus supporting the use of surfaceome screen as an alternative screening strategy. “ (P7, L41-47) is a very lenient interpretation of the data in Fig S4C, for which a less sanguine interpretation might be that the disparate results from the genome-wide and surfaceome screens means that either or both is giving a very noisy readout beyond the top one or two hits. The authors acknowledge this in the following paragraph: “Except for ICAM-1, these screens resulted in no overlap in the top 10 hits”

This philosophical leniency is pronounced in Fig 2, where it is clear that ICAM-1 and RAB5C are consistent hits across a variety of assays, but the authors continue to argue that the other genes in these lists are also hits. I would encourage the authors to lean toward effect size, rather than P-value, as the guiding metric for whether a hit is important or not. Also, ATP6AP1 is indeed an essential gene so it is not surprising that isogenic knockouts could not be grown.

Figure 3A – RAB5C is not visible on these plots. Is it overlaid with ICAM-1? Once again, OLFML3 is a weak hit, with trivial effect size in 3e,f,g,h,I,j, and far weaker. Fig 3J is particularly egregious. On the other hand, kudos to the authors for being forthright about the lack of effect on RV attachment or entry (p11-12, Fig S9).

The RNA-seq analysis of P13 (Fig S10) is nonsensical. The Viral RNA% of FigS10B is potentially interesting, if well controlled, but the differential expression analysis is entirely about genes differentially expressed in mock vs. B14 infection. As for control in S10B, why is the viral RNA% of nontargeting B14-exposed cells 40%, but only 30% in the same experiment in S11B? And why are the six samples in the nontargeting columns of Fig S10D not identical, in fact not even close, to the same columns in Fig S11D? It is literally the same experiment. It is this lack of consistency among controls and weak effect sizes of, especially, OLFML3, that cause this author to doubt many of the statements proposed here.

Bottom line: the conclusions of this paper are not supported by the data. Focus on effect sizes and consistencies in your data, and don’t assume inconsistencies are biologically interesting.

**Authors Response**

**Point-by-point responses to the reviewers’ comments:**

Reviewer #1: They have provided the evidence or results and made a revised manuscript accordingly.

**Response: We appreciate the reviewer’s comments.**

Reviewer #2: Mei et al considerably revise this work. There is a much improved quality control of the CRISPR screens, suggesting that their screens do provide some interesting information. This statement: “we found that genome-wide and surfaceome screens identified different sets of hit genes for surface proteins (Additional file 1: Fig. S4c), thus supporting the use of surfaceome screen as an alternative screening strategy. “ (P7, L41-47) is a very lenient interpretation of the data in Fig S4C, for which a less sanguine interpretation might be that the disparate results from the genome-wide and surfaceome screens means that either or both is giving a very noisy readout beyond the top one or two hits. The authors acknowledge this in the following paragraph: “Except for ICAM-1, these screens resulted in no overlap in the top 10 hits”

**Response: We thank the reviewer for appreciating the improved quality of the manuscript. However, we do not agree with the reviewer’s interpretation of the difference between surfaceome screen and genome-wide screen as the “noise” in the screens. For reason one, we performed appropriate control, comparison and pseudo screen to demonstrate that the different hits between the two screens were real. The reviewer seems to be satisfied with our control experiment in the last revision. For reason two, it is not unusual to see different hits from different CRISPR libraries because the hit identification depends on numerous factors, including the selection of the gene list, design of sgRNA and analysis algorithm. In fact, focused CRISPR screen has been widely used and is known to have advantages for certain applications.**

This philosophical leniency is pronounced in Fig 2, where it is clear that ICAM-1 and RAB5C are consistent hits across a variety of assays, but the authors continue to argue that the other genes in these lists are also hits. I would encourage the authors to lean toward effect size, rather than P-value, as the guiding metric for whether a hit is important or not. Also, ATP6AP1 is indeed an essential gene so it is not surprising that isogenic knockouts could not be grown.

**Response: We thank the reviewer for acknowledging our results and interpretation on ATP6AP1. There were other hits identified from the surfaceome screen and the phenotypic experiments using polyclones or single clones on viral titers, infection rates and cytopathic effects (CPEs) all supported our conclusion. With regard to the effect size, most existing CRISPR screen studies have used P value as the approach to identifying hits, with subsequent validation experiments. Our studies in fact follow this “gold standard” of CRISPR screen. Effect size has higher priority to P value as the measuring metrics only for specific experiments, such as when the performance of a certain gene need to be compared across multiple screens (Emmer *et al.*, 2021, *PLOS Genetics*) or when screening data is used to pre-compute sgRNA efficiencies (Allen *et al.*, 2019, *Genome Res*). To fully address this concern, we have also re-analyzed the MAGeCK results for the top 10 hits from surfaceome screen (see “File for Review Only”). The results confirmed that all the top 10 hits have log2(fold change) values of more than or close to 1, indicating of consistent results using effect size.**

Figure 3A – RAB5C is not visible on these plots. Is it overlaid with ICAM-1? Once again, OLFML3 is a weak hit, with trivial effect size in 3e,f,g,h,I,j, and far weaker. Fig 3J is particularly egregious. On the other hand, kudos to the authors for being forthright about the lack of effect on RV attachment or entry (p11-12, Fig S9).

**Response: We have re-drawn Fig. 3a-b so that RAB5C is clearly visible (and yes it is overlaid with ICAM-1). With regard to the phenotype of OLFML3, we have to admit that we were not anticipating to identify new receptors of RVs because RVs are well characterized viruses that have been studied for decades. As knockout of viral receptors will block viral entry and all the subsequent process, it is expected that ICAM-1 KO will have the strongest phenotype. In other words, one should not expect OLFML3 to have strongest phenotype for all experiments. Particularly, we have demonstrated that OLFML3 involves in a specific signaling pathway in type I IFN signaling. In fact, from Fig. 3e-j, one can easily tell that OLFML3 KO has significant difference from mock group, so as from Fig. 6 for the change of signaling.**

The RNA-seq analysis of P13 (Fig S10) is nonsensical. The Viral RNA% of FigS10B is potentially interesting, if well controlled, but the differential expression analysis is entirely about genes differentially expressed in mock vs. B14 infection. As for control in S10B, why is the viral RNA% of nontargeting B14-exposed cells 40%, but only 30% in the same experiment in S11B? And why are the six samples in the nontargeting columns of Fig S10D not identical, in fact not even close, to the same columns in Fig S11D? It is literally the same experiment. It is this lack of consistency among controls and weak effect sizes of, especially, OLFML3, that cause this author to doubt many of the statements proposed here.

**Response: We disagree with the reviewer’s interpretation on the RNA-Seq data. First of all, we have clearly stated how the DEG is calculated in the legend of Fig. S10 as “RV-induced perturbation of gene expression is first analyzed and the differentially upregulated or downregulated genes between mock and knockout cells are defined as DEGs”. Therefore, the reviewer’s comment “but the differential expression analysis is entirely about genes differentially expressed in mock vs. B14 infection” is wrong. Second, the data in Fig. S10 and S11 were from two separate experiments. We believe that the difference between the %viral RNA of 40% and 30% is within the acceptable range from the perspective of virology. This is because these data were the NGS of infected cells and many experimental factors may contribute to the final outcome. One should not expect identical results from two separate experiments. Third, with regard to the pattern of column, we have clearly indicated that the data are presented as Z scores, which normalize gene expression within each gene entry (each row in the heat map). Therefore, one should not use Z score to compare across rows, nor even across different experiments.**

Bottom line: the conclusions of this paper are not supported by the data. Focus on effect sizes and consistencies in your data, and don’t assume inconsistencies are biologically interesting.

**Response: We do not agree with the reviewer’s conclusion and believe that the conclusion of our study is supported by the data and that the experiments are generally consistent.**
