# Supplementary material for: Correction: Surfaceome CRISPR screen identifies OLFML3 as a rhinovirus-inducible IFN antagonist
Source: Genome Biol. 2021 Nov 15;22:314. doi: 10.1186/s13059-021-02534-5 (PMC8591863; doi:10.1186/s13059-021-02534-5)
Supplement: Supplementary file 1 — Additional file 1: Fig. S1. Construction of CRISPR genome-wide and surfaceome libraries. Fig. S2. Quality analyses of constructed genome-wide and surfaceome CRISPR libraries. Fig. S3. Evaluation of surfaceome and genome-wide CRISPR libraries. Fig. S4. Validation of the screening results. Fig. S5. Determination of gene modification efficiency. Fig. S6. Validation of the top 10 hits from surfaceome and genome-wide screens. Fig. S7. Construction and validation of single clones of ICAM-1−/−, RAB5C−/−, OLFML3−/−, SLC4A7−/− and ATP6AP1−/+ H1-Hela cells. Fig. S8. Validation of the effects of ICAM-1, RAB5C and OLFML3 on RV infection, related to Fig. 3. Fig. S9. Dissection of the functions of RAB5C and OLFML3 in RV infection. Fig. S10. RNA-seq analyses of the effects of RAB5C knockout on RV infection. Fig. S11. RNA-Seq analyses of the effects of OLFML3 on RV infection (related to Fig. 4). Fig. S12. Bar plots showing RT-qPCR quantification of ISG expression in mock and OLML3−/− cells at 24 h post infection of RV-B14 (a) and RV-A16 (b) at an MOI of 2 [file 13059_2021_2534_MOESM1_ESM.docx]

**Additional file 1: Supplementary figures**

**Surfaceome CRISPR screen identifies OLFML3 as a rhinovirus-inducible IFN antagonist**

Hong Mei^1, †^, Zhao Zha^1, †^, Wei Wang^1, †^, Yusang Xie^2^, Yuege Huang^1,3^, Wenping Li^1,3^, Dong Wei^4^, Xinxin Zhang^4^, Jieming Qu^2,^ *, Jia Liu^1,5,6,7,8^*

^1^Shanghai Institute for Advanced Immunochemical Studies and School of Life Science and Technology, ShanghaiTech University, Shanghai, 201210, P. R. China

^2^Department of Respiratory and Critical Care Medicine, Ruijin Hospital and Institutes of Respiratory Diseases, School of Medicine, Shanghai Jiao Tong University, Shanghai 200025, China

^3^University of Chinese Academy of Sciences, Beijing, 100049, China

^4^Research Laboratory of Clinical Virology, Ruijin Hospital, Shanghai Jiaotong University School of Medicine, Shanghai 200025, China

^5^Shanghai Clinical Research and Trial Center, Shanghai, 201210, P. R. China

^6^State Key Laboratory of Respiratory Disease, Guangzhou Medical University, Guangzhou, 510182, Guangdong Province, China

^7^Gene Editing Center, School of Life Science and Technology, ShanghaiTech University, 201210, P. R. China

^8^Guangzhou Laboratory, No. 9 XingDaoHuanBei Road, Guangzhou International Bio Island, Guangzhou 510005, Guangdong Province, China

^†^These authors contributed equally to this work

Correspondence should be addressed to J.L. (liujia@shanghaitech.edu.cn), J.Q. (jmqu0906@163.com)

**Additional file1: Fig S1-S10**

**Table of Content:**

Fig. S1. Construction of CRISPR genome-wide and surfaceome libraries.

Fig. S2. Quality analyses of constructed genome-wide and surfaceome CRISPR libraries.

Fig. S3. Evaluation of surfaceome and genome-wide CRISPR libraries.

Fig. S4. Validation of the screening results.

Fig. S5. Determination of gene modification efficiency.

Fig. S6. Validation of the top 10 hits from surfaceome and genome-wide screens.

Fig. S7. Construction and validation of single clones of ICAM-1^-/-^, RAB5C^-/-^, OLFML3^-/-^, SLC4A7^-/-^ and ATP6AP1^-/+^ H1-Hela cells.

Fig. S8. Validation of the effects of ICAM-1, RAB5C and OLFML3 on RV infection, related to Fig. 3.

Fig. S9. Dissection of the functions of RAB5C and OLFML3 in RV infection.4

Fig. S10. RNA-seq analyses of the effects of RAB5C knockout on RV infection.

Fig. S11. RNA-Seq analyses of the effects of OLFML3 on RV infection (related to Fig. 4).

Fig. S12. Bar plots showing RT-qPCR quantification of ISG expression in mock and OLML3-/- cells at 24 h post infection of RV-B14 (a) and RV-A16 (b) at an MOI of 2.

**
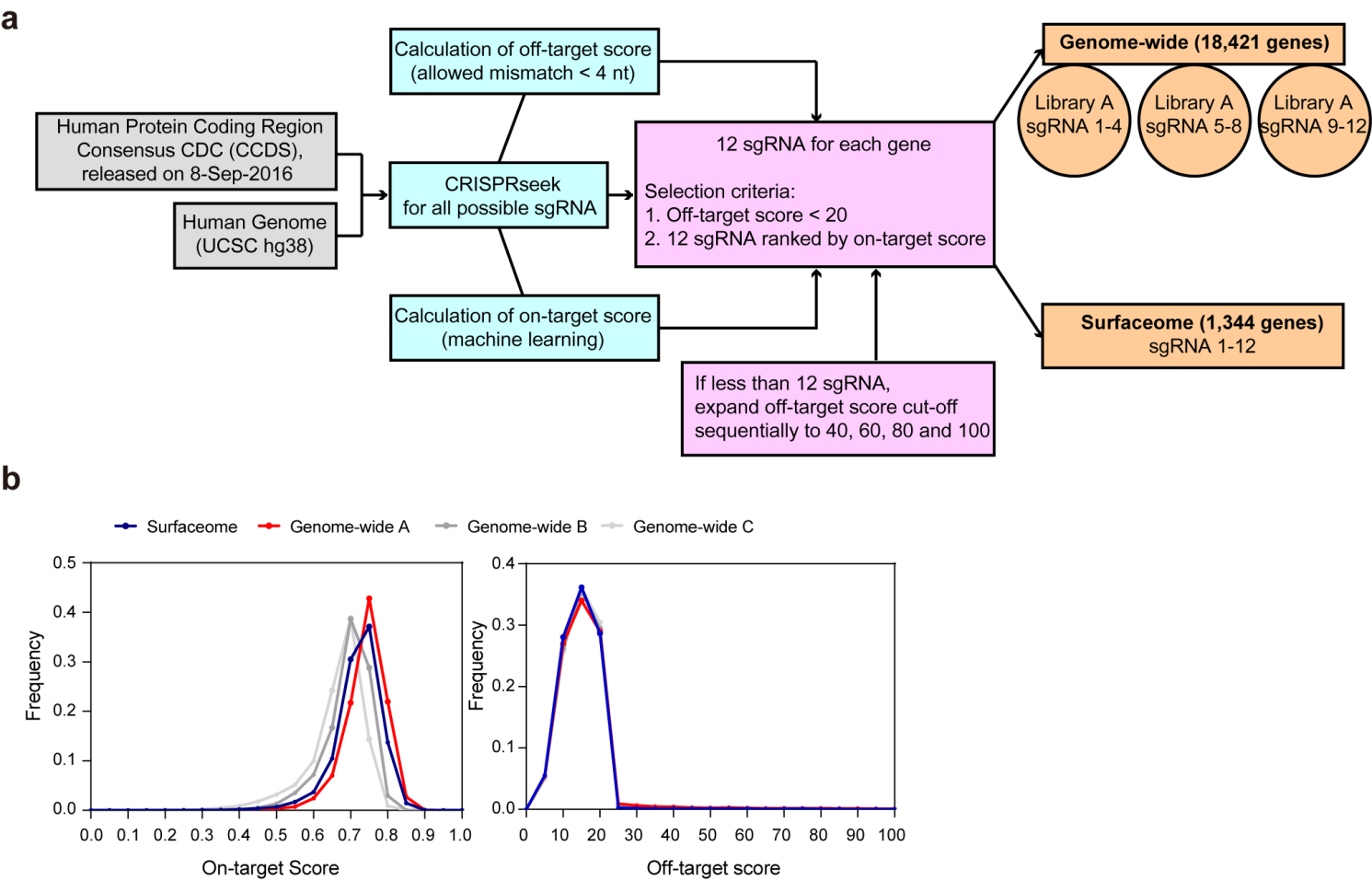
**

**Fig. S1 Construction of CRISPR genome-wide and surfaceome libraries. a** Schematic illustration. **b** The distribution of sgRNA on-target and off-target scores in genome-wide sub-libraries A, B, C and surfaceome library.

**
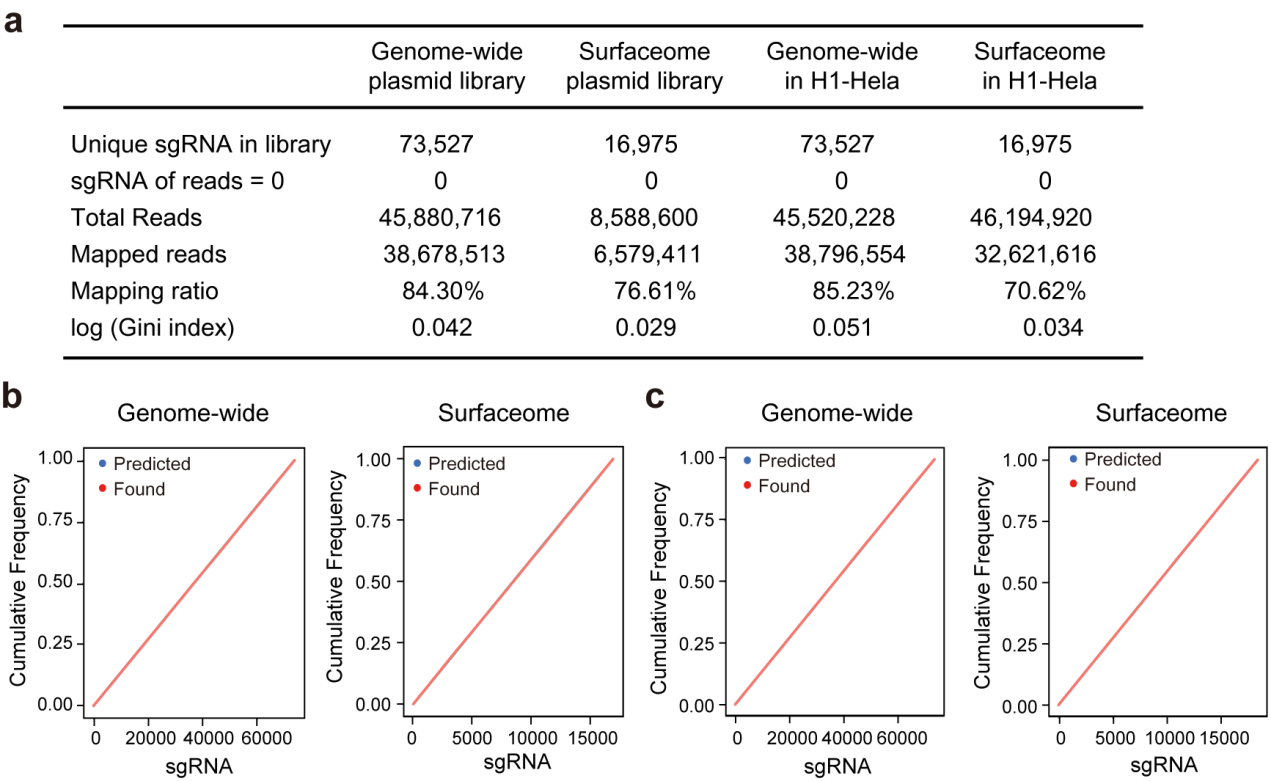
**

**Fig. S2 Quality analyses of constructed genome-wide and surfaceome CRISPR libraries.** **a** Summary of next-generation sequencing results of sgRNA in the genome-wide and surfaceome plasmid and H1-Hela libraries. **b**-**c** Distribution of sgRNA in genome-wide and surfaceome libraries in pooled plasmids (**b**) and H1-Hela cells (**c**).


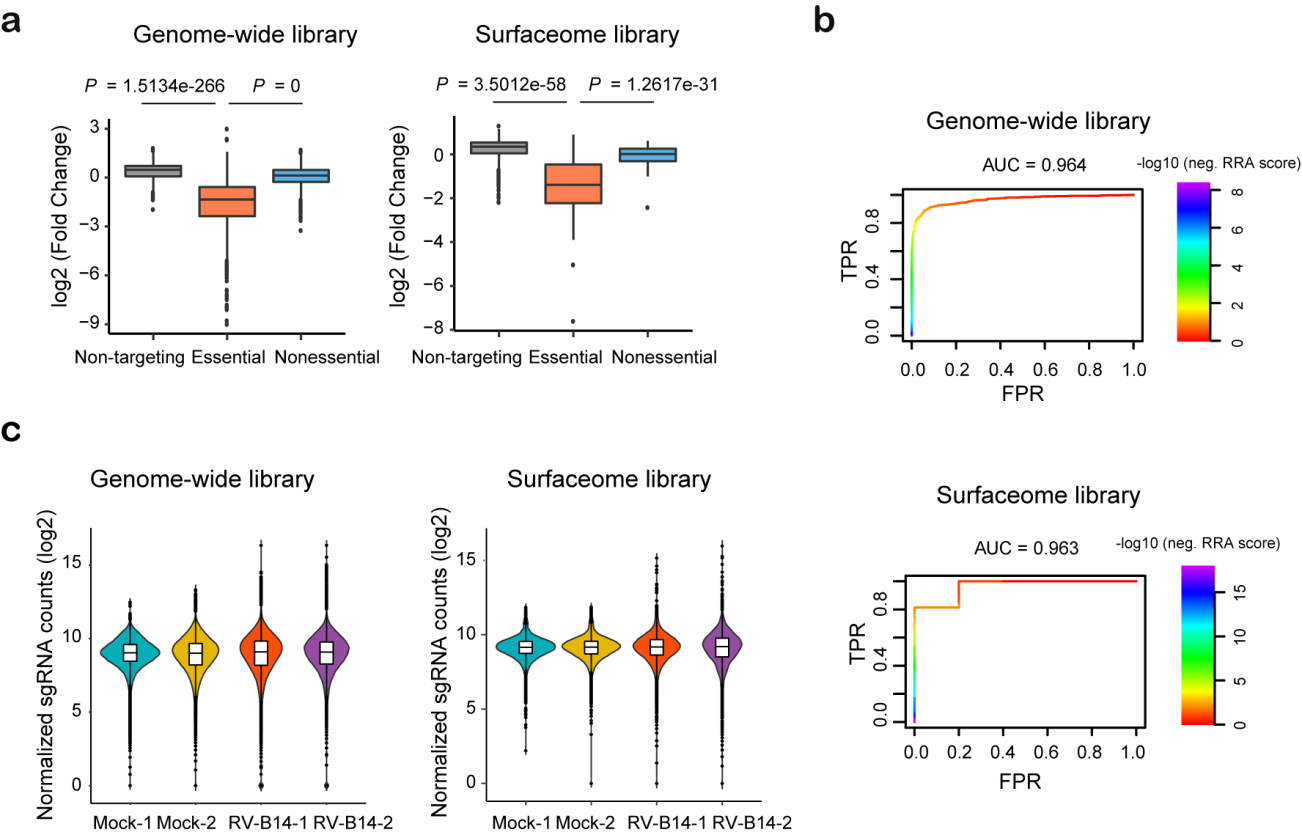


**Fig. S3** **Evaluation of** **surfaceome and genome-wide CRISPR libraries.** **a** The fold change distribution of non-targeting sgRNAs and sgRNAs targeting essential genes and nonessential genes in genome-wide and surfaceome library. Statistical analysis is performed using Student’s *t* test. **b** Receiver operating characteristic (ROC) curves showing the classification performance of essential and non-essential genes in genome-wide and surfaceome libraries. In total, 684 essential and 927 non-essential genes were included in the genome-wide library, and 27 essential and 7 non-essential genes were included in the surfaceome library. ROC curves are drawn by R package PRROC according to the a-RRA scores. True positive rate (TPR) is plotted with false positive rate (FPR). **c** SgRNA distribution in surfaceome and genome-wide CRISPR libraries post RV-B14 challenge.


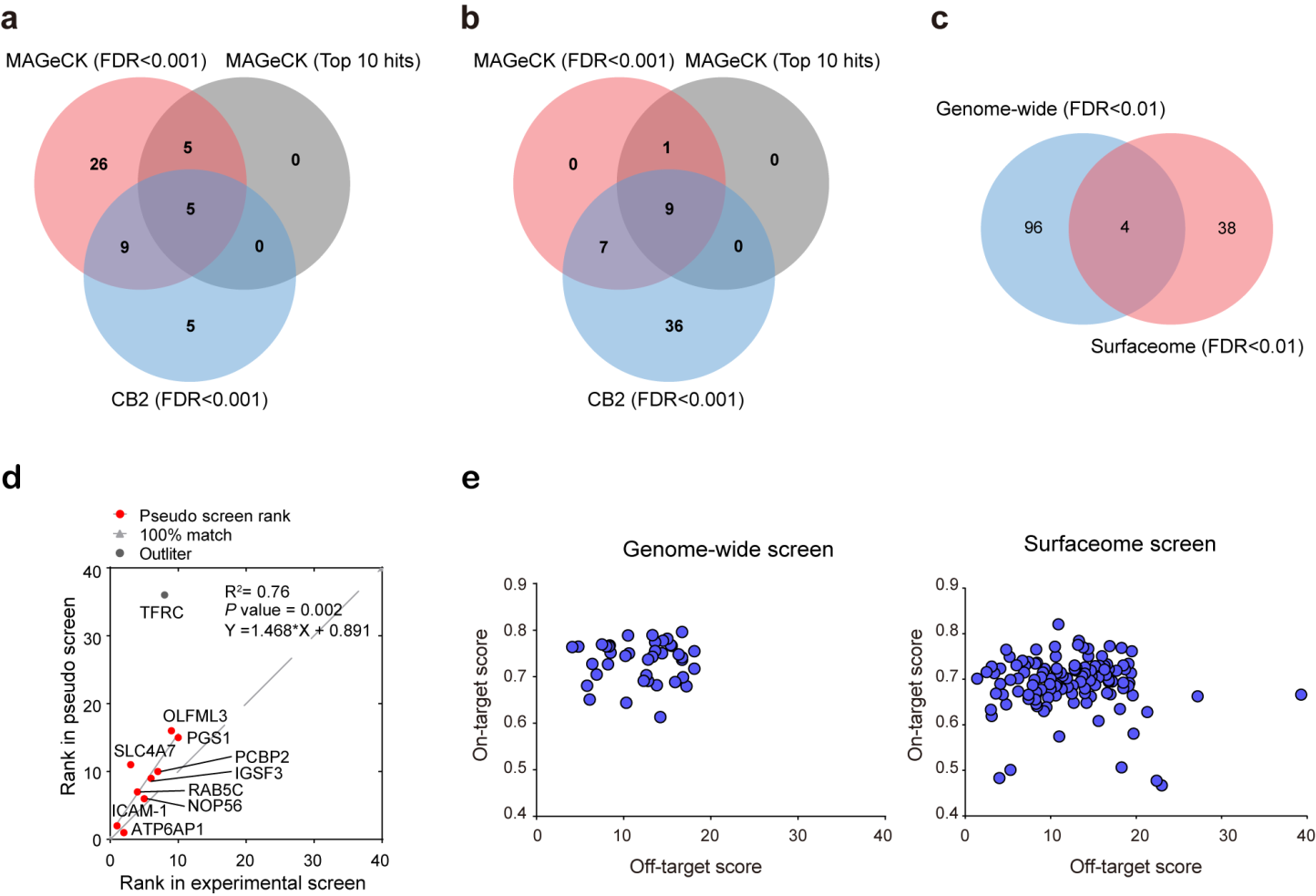


**Fig. S4 Validation of the screening results.** **a-b**, Venn diagram showing the overlaps between the genes identified by MAGeCK (FDR<0.001), CB^2^ (FDR<0.001) and the top 10 hits from MAGeCK for genome-wide (a) and surfaceome screens (b). **c** Venn diagram showing the overlaps between the surface genes identified from genome-wide and surfaceome screens by MAGeCK analysis using an FDR cut-off of 0.01. **d** Correlation analyses of the top 10 hits from experimental surfaceome screen and their ranks in *in silico* (pseudo) surfaceome screen using sgRNA 1-4. The R square and *P* values of the correlation are calculated and shown. **e** Scatter plot showing the on-target and off-target scores of sgRNAs of the top 10 hit genes from genome-wide and surfaceome screens respectively.


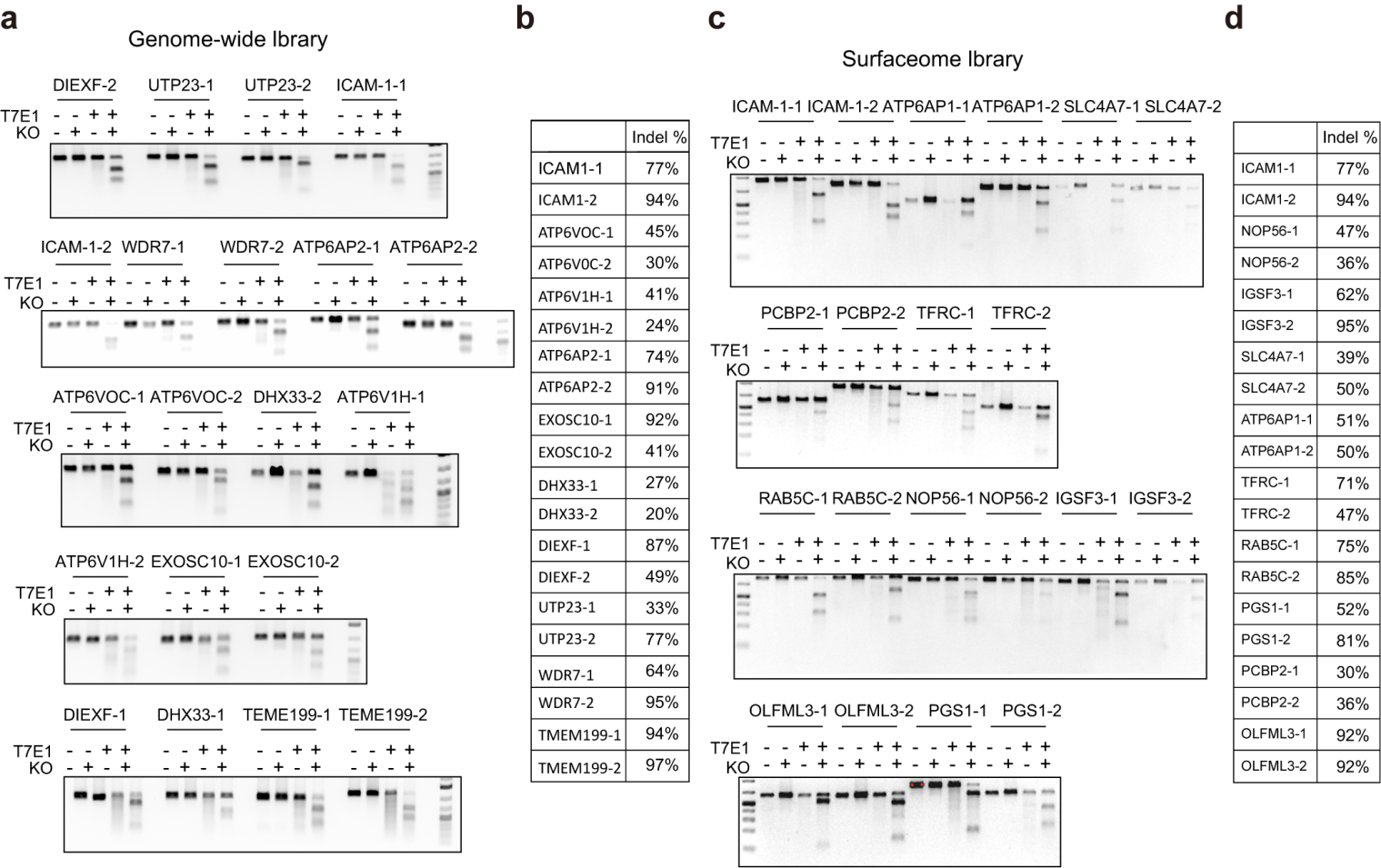


**Fig. S5 Determination of gene modification efficiency.** Knockout of genes identified from surfaceome (a-b) and genome-wide (c-d) libraries were evaluated with T7E1 (a, c) and Sanger sequencing (b, d) respectively.


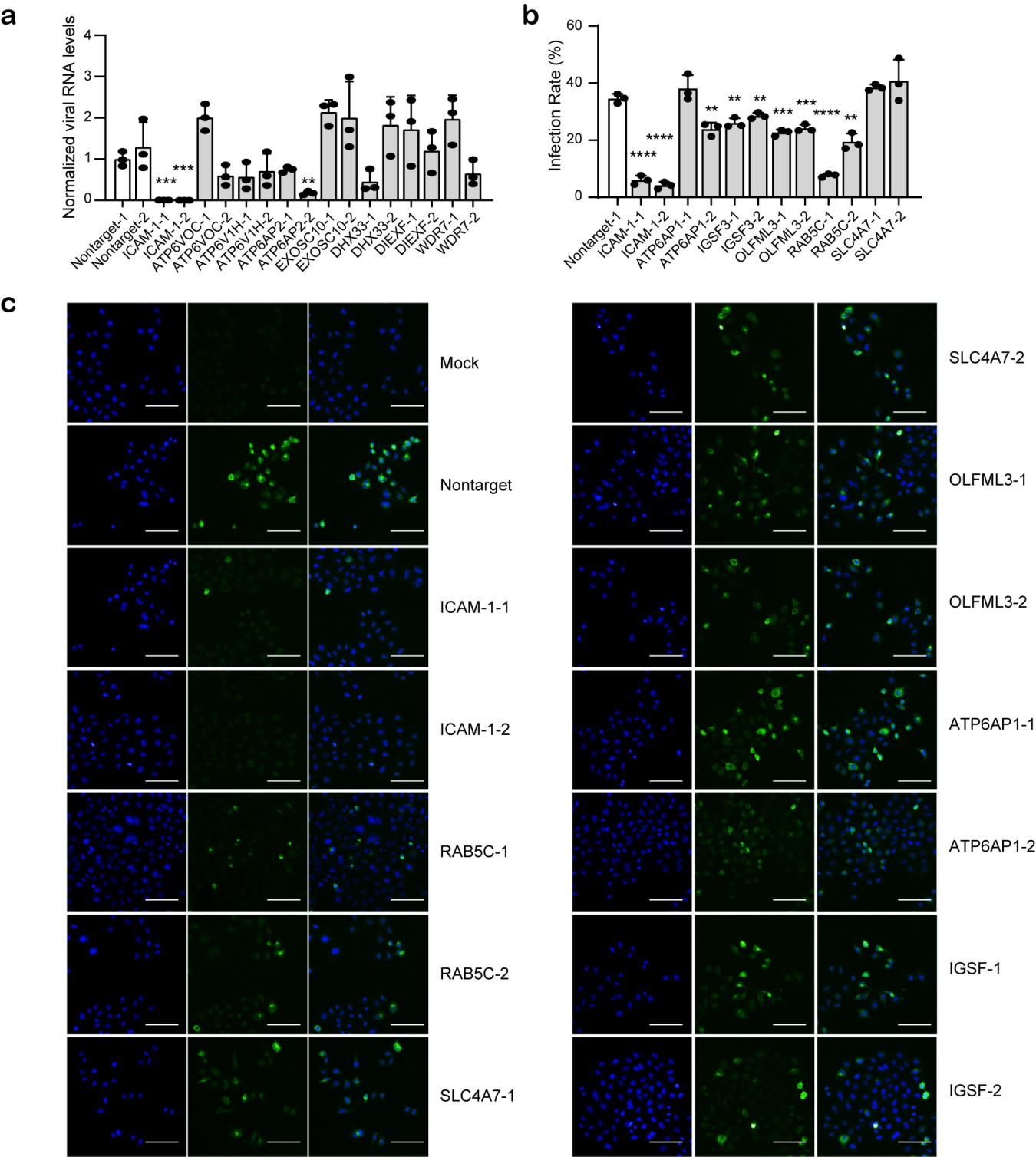


**Fig. S6 Validation of the top 10 hits from surfaceome and genome-wide screens. a** RT-qPCR quantification of viral loads in the lysate of knockout cells of the top 10 hits identified from genome-wide screen. Cell lysate is harvested at 24 h post RV-B14 infection at an MOI of 2. Viral RNA is normalized to RPLP0. **b-c** Immunofluorescence (IF) staining of RV-B14 envelope protein in infected knockout cells. IF staining is performed at 16 h post infection of RV-B14 at an MOI of 1. **b** Quantification of RV-B14 envelope protein. Each biological replicate contains the quantification results from 2,000 cells. **c** Representative IF images of mock and knockout cells. DAPI, blue; RV-B14 envelope protein, green. Scale bar, 100 μm. The significant difference between mock and knockout cells is determined using two-tailed Student’s *t*-test, *, *P* <0.05; **, *P* <0.01; ***, *P* <0.001; ****, *P* <0.0001.

**
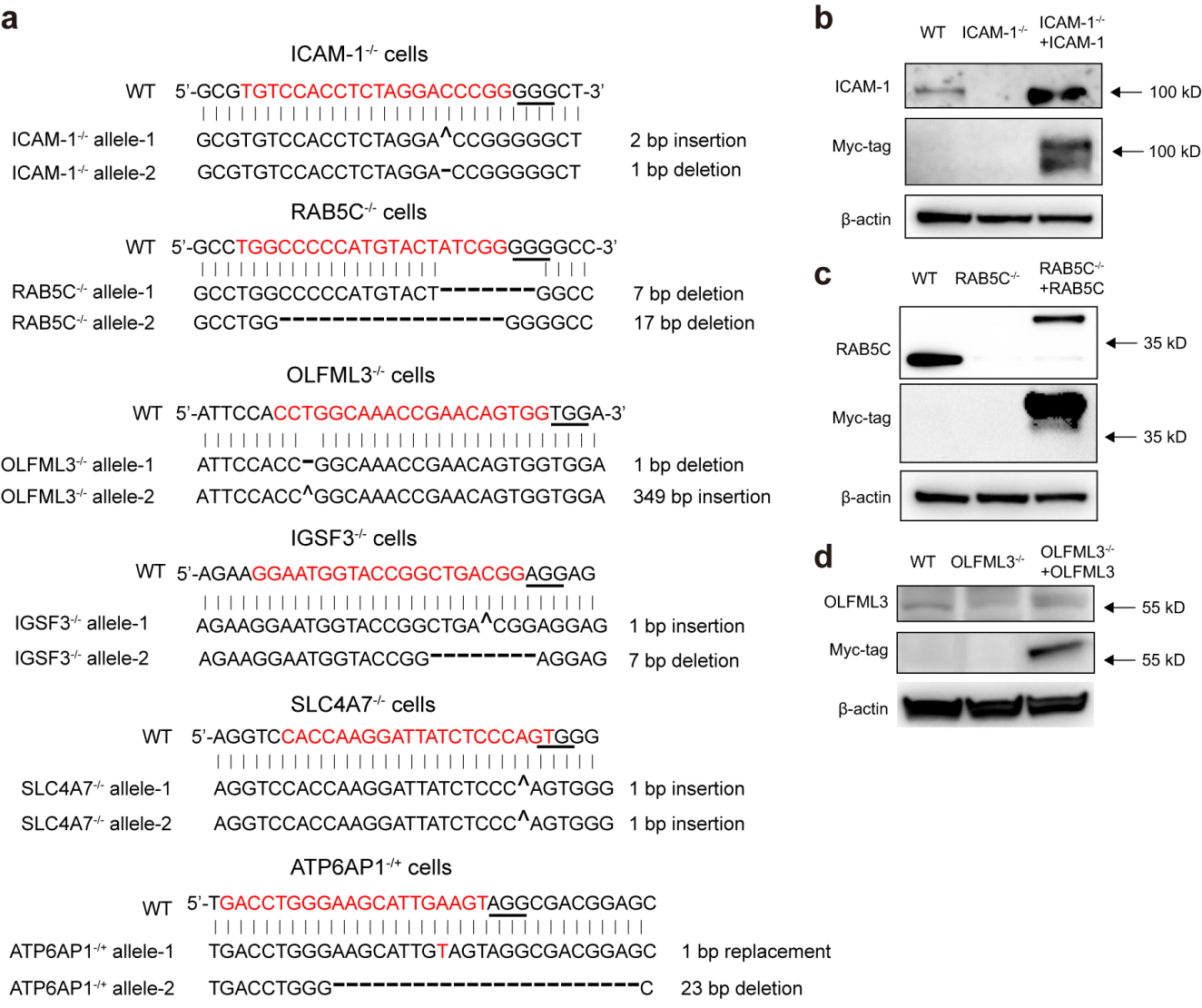
**

**Fig. S7 Construction and validation of single clones of ICAM-1^-/-^, RAB5C^-/-^, OLFML3^-/-^, SLC4A7^-/-^ and ATP6AP1^-/+^ H1-Hela cells. a** Sanger sequencing analyses of mutated alleles. The 20-bp CRISPR-Cas9 target sequences are highlighted in red and protospacer adjacent motif (PAM) underlined. **b-d** Western blot analyses of ICAM-1 (**b**), RAB5C (**c**) and OLFML3 (**d**) expression in wide-type, knockout and rescued cells.


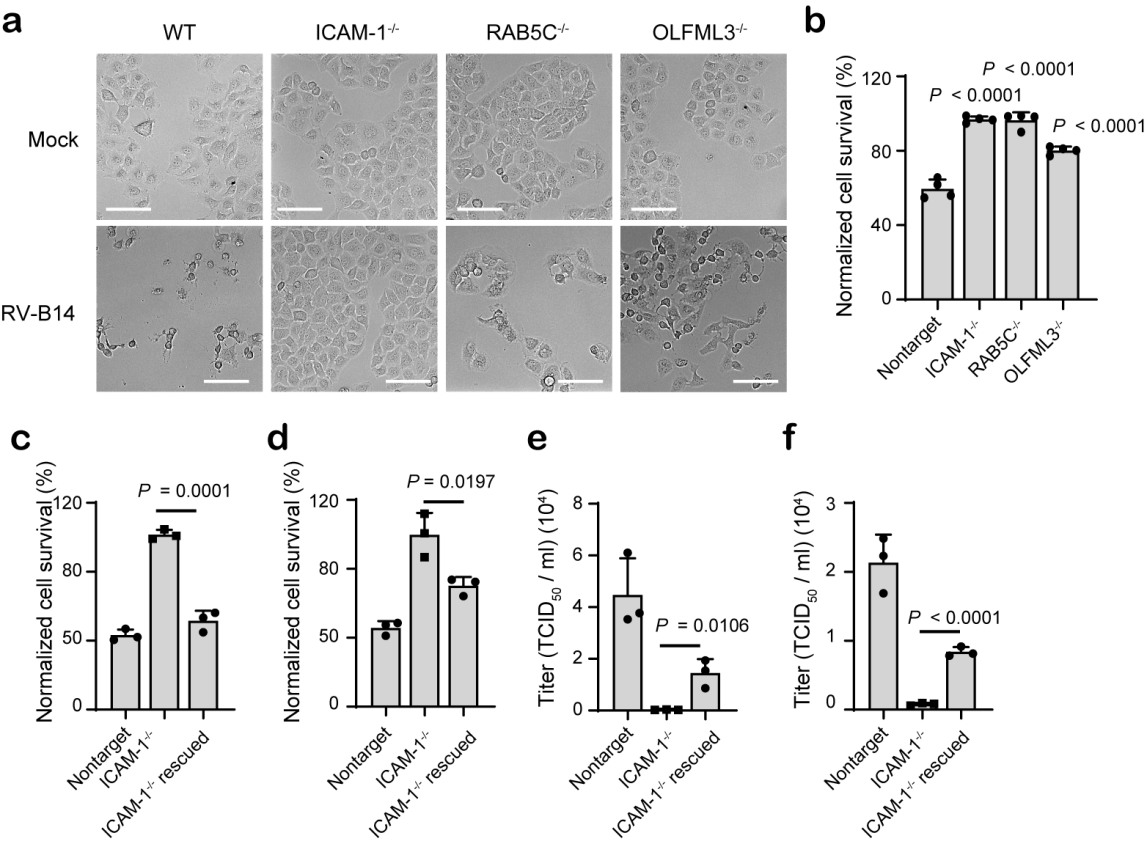


**Fig. S8 Validation of the effects of ICAM-1, RAB5C and OLFML3 on RV infection, related to Fig. 3. a**-**b** Cell viability assay to determine the protective effects of ICAM-1, RAB5C and OLFML3 knockout against RV-B14 infection. Experiments are performed with an MOI of 2 and cell viability is determined at 24 h post infection. **a** Representative images. Scale bar, 100 μm. **b** Quantification of cell viability. Significant difference between test groups and non-targeting sgRNA group is determined using two-tailed Student’s *T* test and the *P* values are shown. **c**-**d** Cell viability assay to determine the effects of ICAM-1 overexpression on RV-B14 (**c**) or RV-A16 (**d**)-induced cell death in ICAM-1^-/-^ cells. **e-f** Rescued susceptibility of ICAM-1^-/-^ H1-Hela cells to RV-B14 (**e**) and RV-A16 (**f**) infection by ICAM-1 overexpression, as determined by viral loads in medium supernatant. Significant difference between knockout and rescued cells is determined using two-tailed unpaired Student’s *t* test.

**
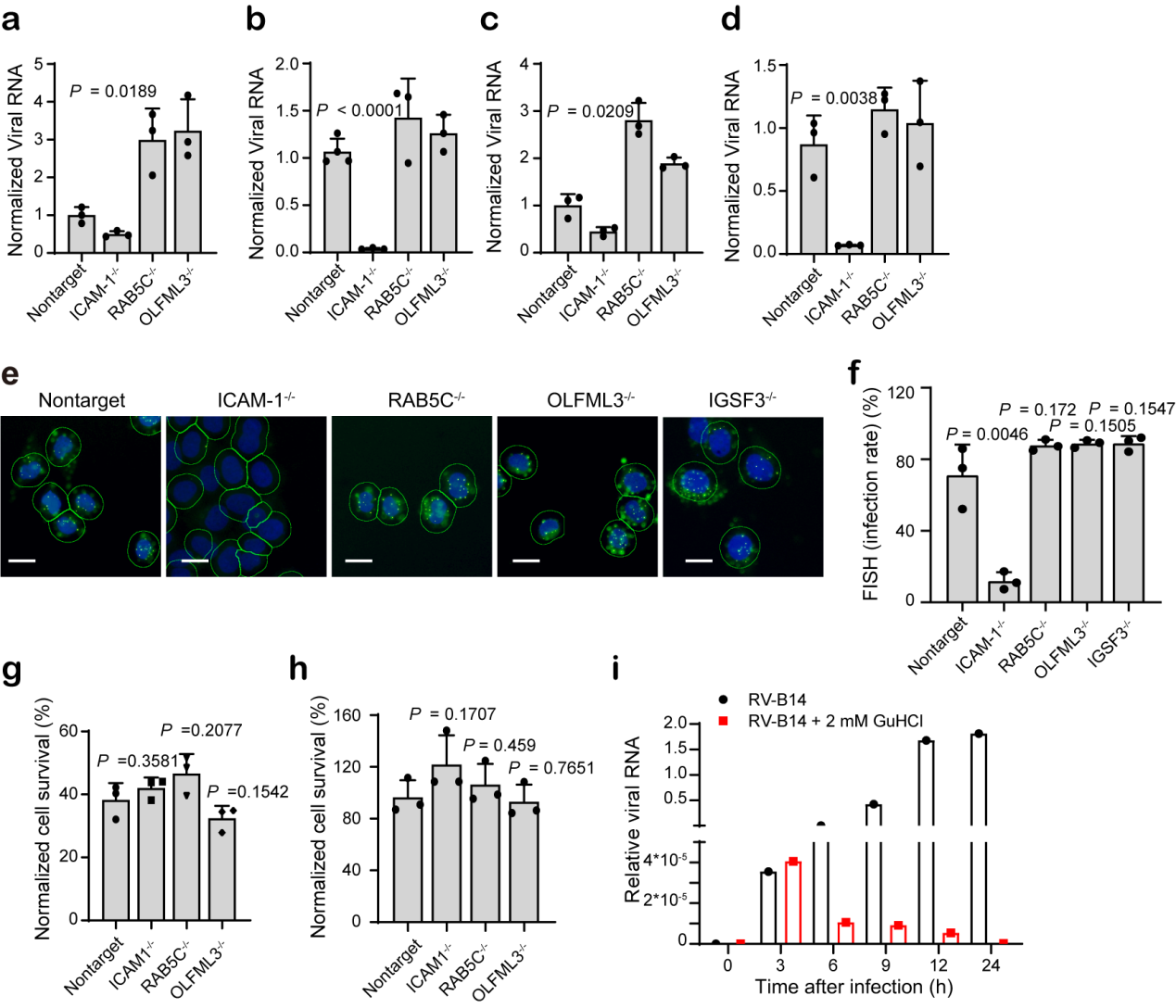
**

**Fig. S9 Dissection of the functions of RAB5C and OLFML3 in RV infection. a-b** RV-B14 attachment (**a**) and entry (**b**) assays. **c**-**d** RV-A16 attachment (c) and entry (d) assays. For **a-d**, attached or internalized RV RNA is normalized to RPLP0. **e** Representative images. DAPI, blue; viral genome RNA, green dots; cell membrane, green lines. Scale bar, 20 μm. **f** Quantification of FISH experiments. The results are shown as mean ± SD (*n* = 3). In each replicate, 5,000 cells are analyzed. **g** Cell viability of mock and knockout cells at 24 h after transfection of RV-A16 genome RNA. **h** Cell viability at 6 h after treatment with 2 mM GuHCl. Significant difference between mock and knockout cells is determined using two-tailed unpaired Student’s *t* test. **i** Inhibition of the synthesis of RV-B14 viral RNA by treatment with 2 mM GuHCl. Cells are infected with RV-B14 at an MOI of 20 and viral RNA in cell lysates is determined and normalized to RPLP0.


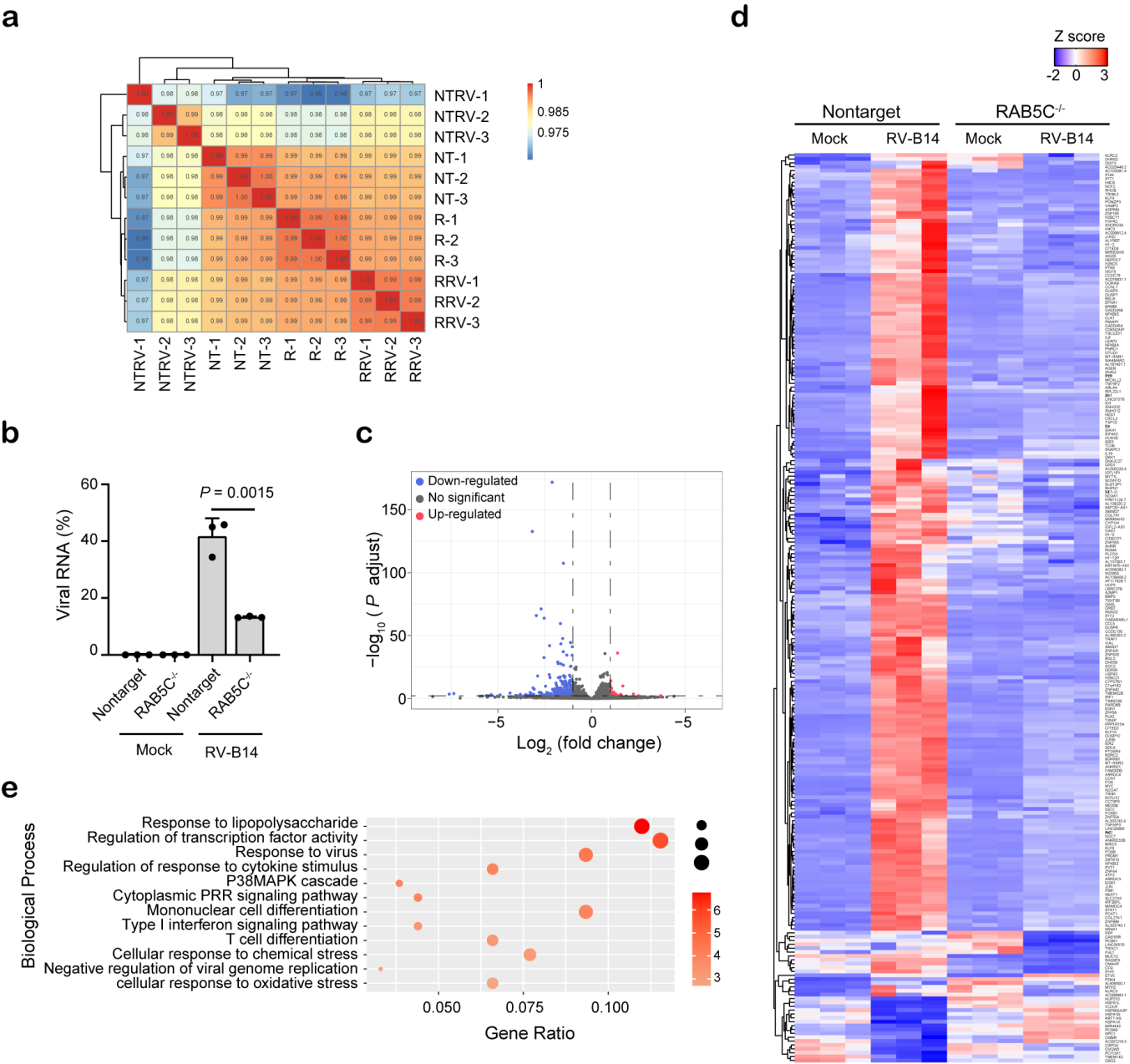


**Fig. S10 RNA-seq analyses of the effects of RAB5C knockout on RV infection. a** Pearson correlation analyses of sequenced samples. NT, non-targeting sgRNA; NTR, non-targeting sgRNA with RV infection; R, RAB5C knockout; RRV, RAB5C knockout with RV infection. Cells are harvested and analyzed at 24 h after infection of RV-B14 at an MOI of 2. **b** Analyses of the effects of RAB5C knockout on the transcriptomic expression of RV-B14. The significant difference of RV transcriptomic expression between mock and RAB5C^-/-^ cells is determined using two-tailed unpaired Student’s *t* test. **c** Volcano plot showing differentially expressed genes (DEGs). RV-induced perturbation of gene expression is first analyzed and the differentially upregulated or downregulated genes between mock and knockout cells are defined as DEGs. **d** Heat map showing the DEGs with adjusted *P* values of less than 0.05 and fold change of more than 2. **e** GO analyses of biological processes of DEGs.

**
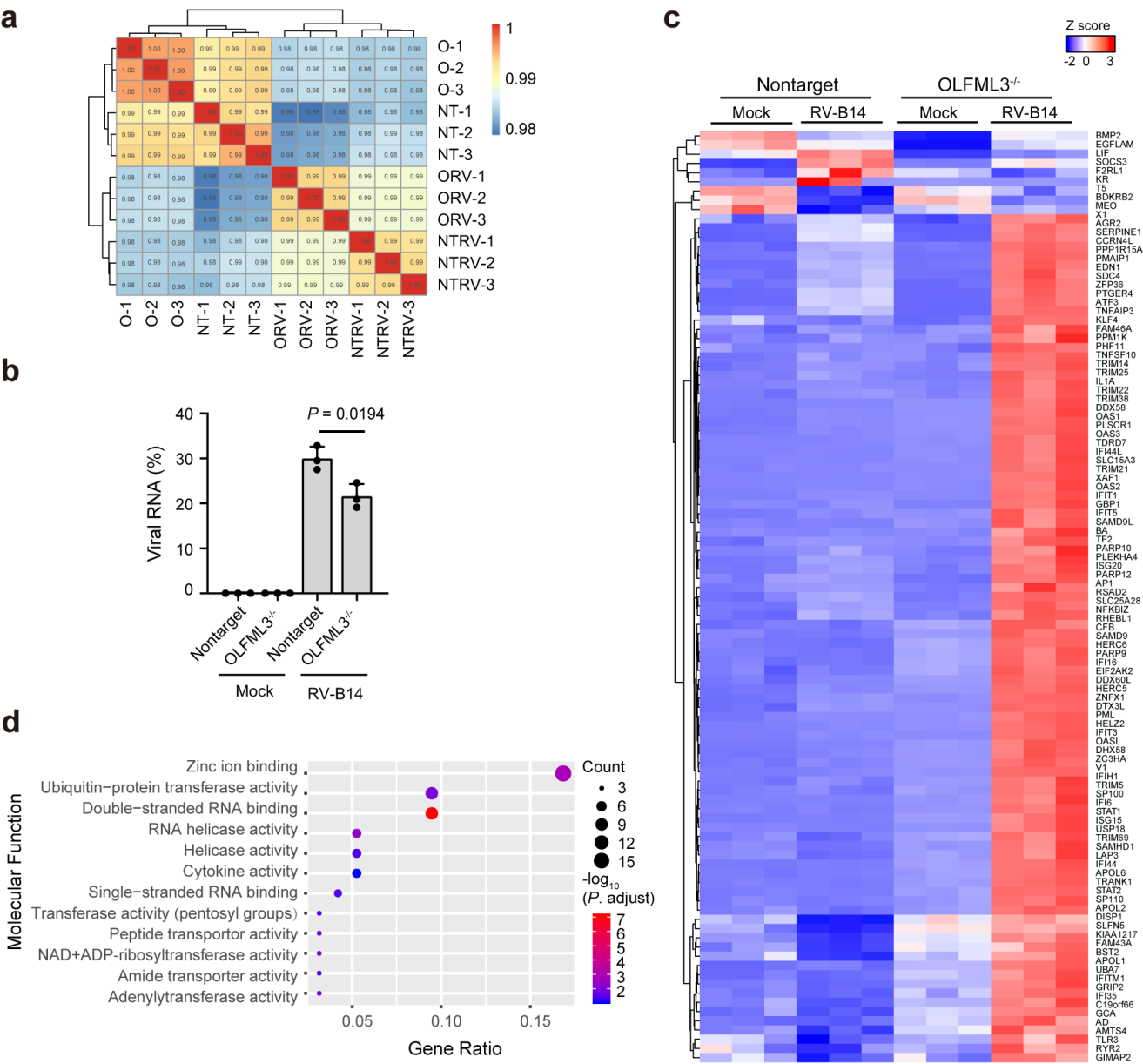
**

**Fig. S11 RNA-Seq analyses of the effects of OLFML3 on RV infection (related to Fig. 4). a** Pearson correlation analyses of sequenced samples. NT, non-targeting sgRNA; NTRV, non-targeting sgRNA with RV infection; O, OLFML3 knockout; ORV, OLFML3 knockout with RV infection. **b** Analyses of the effects of OLFML3 knockout on the transcriptomic expression of RV-B14. The significant difference of RV transcriptomic expression between mock and OLFML3^-/-^ cells is determined using two-tailed unpaired Student’s *t* test. **c** Heat map showing the differentially expressed genes with adjusted *P* values of less than 0.05and fold change of more than 2. Cells are collected for RNA-Seq analyses at 24 h after infection with RV-B14 at an MOI of 2. **d** GO analyses of the molecular functions of DEGs.


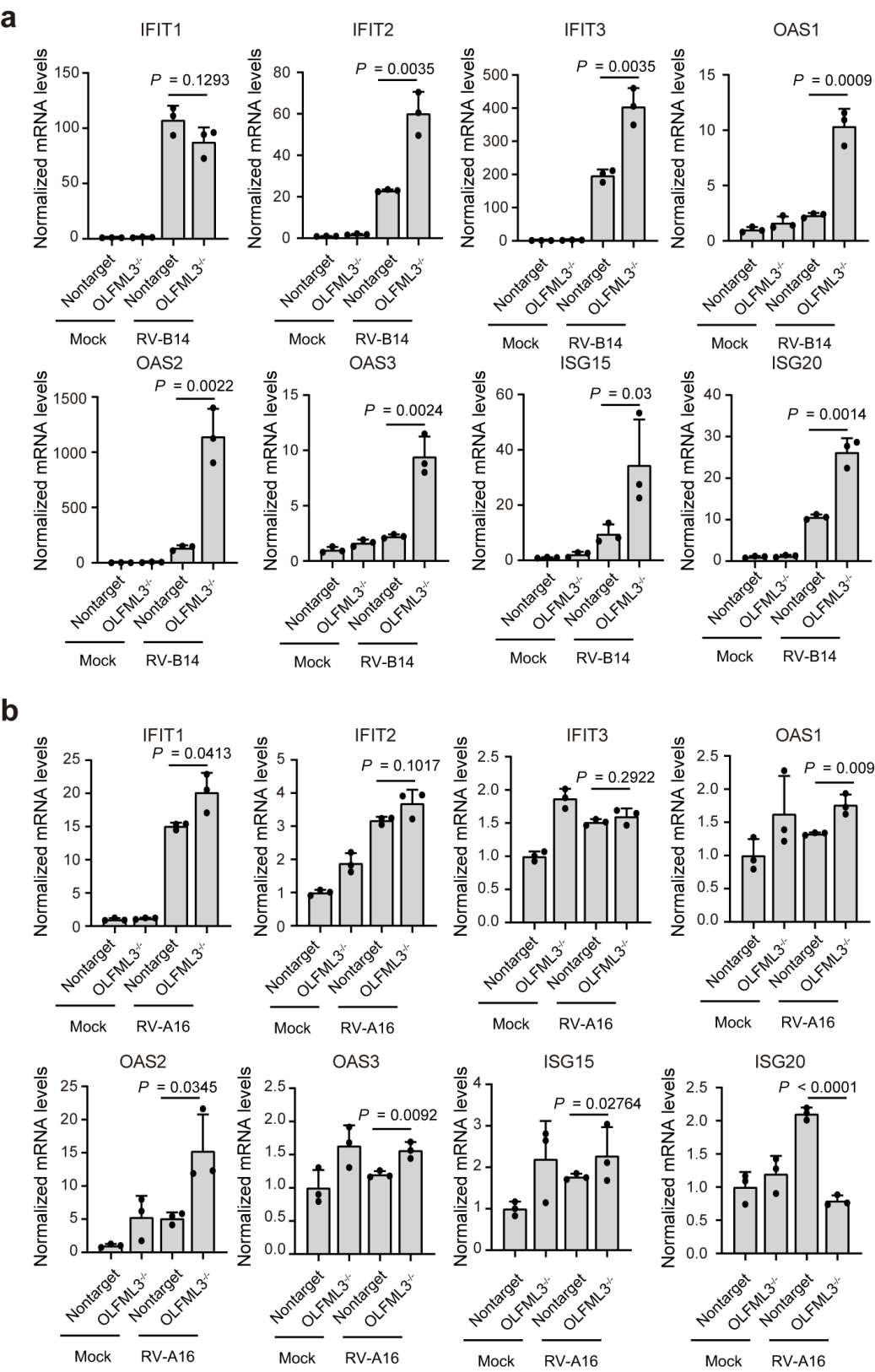


**Fig. S12 Bar plots showing RT-qPCR quantification of ISG expression in mock and OLML3^-/-^ H1-Hela cells at 24 h post infection of RV-B14 (a) and RV-A16 (b) at an MOI of 2.** Gene expression is normalized to non-targeting sgRNA mock group. Significant difference between mock-RV and OLFML3^-/-^-RV is determined using two-tailed Student’s *t* test.
